# Vagus Nerve Stimulation in Ischemic Stroke: Old Wine in a New Bottle

**DOI:** 10.3389/fneur.2014.00107

**Published:** 2014-06-24

**Authors:** Peter Y. Cai, Aakash Bodhit, Roselle Derequito, Saeed Ansari, Fawzi Abukhalil, Spandana Thenkabail, Sarah Ganji, Pradeepan Saravanapavan, Chandana C. Shekar, Sharatchandra Bidari, Michael F. Waters, Vishnumurthy Shushrutha Hedna

**Affiliations:** ^1^Department of Neurology, University of Florida, Gainesville, FL, USA; ^2^Department of Anesthesiology, University of Florida, Gainesville, FL, USA; ^3^Department of Surgery, University of Florida, Gainesville, FL, USA; ^4^Department of Radiology, University of Florida, Gainesville, FL, USA; ^5^Department of Neuroscience, University of Florida, Gainesville, FL, USA

**Keywords:** stroke, middle cerebral artery occlusion, glutamate excitotoxicity, neuroinflammation, cerebral blood flow

## Abstract

Vagus nerve stimulation (VNS) is currently Food and Drug Administration-approved for treatment of both medically refractory partial-onset seizures and severe, recurrent refractory depression, which has failed to respond to medical interventions. Because of its ability to regulate mechanisms well-studied in neuroscience, such as norepinephrine and serotonin release, the vagus nerve may play an important role in regulating cerebral blood flow, edema, inflammation, glutamate excitotoxicity, and neurotrophic processes. There is strong evidence that these same processes are important in stroke pathophysiology. We reviewed the literature for the role of VNS in improving ischemic stroke outcomes by performing a systematic search for publications in Medline (1966–2014) with keywords “VNS AND stroke” in subject headings and key words with no language restrictions. Of the 73 publications retrieved, we identified 7 studies from 3 different research groups that met our final inclusion criteria of research studies addressing the role of VNS in ischemic stroke. Results from these studies suggest that VNS has promising efficacy in reducing stroke volume and attenuating neurological deficits in ischemic stroke models. Given the lack of success in Phase III trials for stroke neuroprotection, it is important to develop new therapies targeting different neuroprotective pathways. Further studies of the possible role of VNS, through normally physiologically active mechanisms, in ischemic stroke therapeutics should be conducted in both animal models and clinical studies. In addition, recent advent of a non-invasive, transcutaneous VNS could provide the potential for easier clinical translation.

## Introduction

### History of vagus nerve stimulation and its application

Since the early half of the twentieth century, experiments showing that vagus nerve stimulation (VNS)-evoked neuronal responses helped investigators study the nucleus tractus solitarius (NTS), the main central nervous system afferent connection of the vagus nerve, and its projections to various cortical structures (1, 2). Initial studies on the effect of VNS on the central nervous system in animal seizure models (dogs, cats, monkeys, rats) all demonstrated beneficial effects of VNS in seizure suppression (3–6). In 1988, the first reported pilot studies for treatment of medically refractory seizures in four patients suggested that VNS had potential for effective seizure control in humans as well (7). In 1997, the U.S. Food and Drug Administration (FDA) approved the use of VNS for treatment of medically refractory partial-onset seizures. In 2005, VNS was approved by the FDA for treatment of severe, recurrent unipolar, and bipolar depression in patients with a history of failed response to at least four antidepressant interventions (8). The potential of VNS to treat partial complex epilepsy, generalized epilepsy, involuntary movement disorders, depression, migraine, and neuropsychiatric disorders has also been proposed (9).

### Vagus nerve stimulation: Anatomy and mechanisms

The vagus nerve, while commonly considered to be a parasympathetic efferent nerve, is composed of about 80% afferent sensory fibers carrying information from the periphery to the brain (10). In the central nervous system, the vagus primarily projects to the NTS and releases excitatory neurotransmitters (glutamate and aspartate), inhibitory neurotransmitter (γ-aminobutyric acid), acetylcholine, and other neuropeptides for signal transduction (11). Subsequently, the NTS has widespread efferent pathways in the central nervous system to the parabrachial nucleus, reticular formation, basal forebrain, amygdala, hippocampus, hypothalamus, dorsal raphe, cerebellum, and spinal cord (12). NTS projections to brainstem nuclei (locus coeruleus and dorsal raphe magnus) modulate serotonin and norepinephrine (NE) release to the entire brain (13). Despite the current level of understanding of vagus nerve anatomy, the mechanisms responsible for VNS treatment efficacy are still poorly understood.

**Figure 1:**
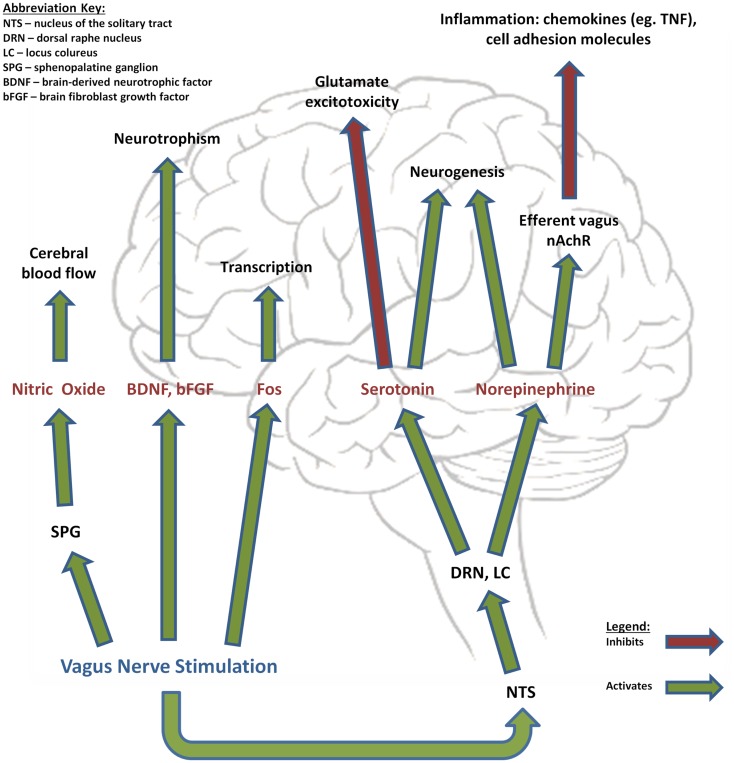
**Effects of vagus nerve stimulation**. Vagus nerve stimulation has been shown to modulate the release of a variety of factors that regulate important mechanisms in stroke pathophysiology, such as cerebral blood flow, neurotrophism, neurogenesis, excitotoxicity, and inflammation.

Acutely stimulating the vagus nerve has been shown to cause activation and deactivation in various regions of the brain, with an increased VNS pulse width producing proportionally more activation than deactivation when compared to a lower pulse width (14). While the final outcome of these changes has not been clearly established, there is experimental evidence for the role of the vagus nerve in regulating a number of distinct physiological pathways: cerebral blood flow (CBF), melanocortins and inflammation, glutamate excitotoxicity, NE, and neurotrophic processes (Figure 1) (15). When utilized in treatment of epilepsy, VNS can be accomplished with a three-component apparatus: (1) a multiprogramable bipolar pulse generator implanted subcutaneously in the left chest wall below the clavicle, (2) two helical electrodes wrapped around the vagus nerve in the cervical area and linked to the pulse generator, and (3) a programing wand linked to software that allows for programing and assessment (12). Individual patients respond best to different combinations of parameter settings and it is the responsibility of the individual physician to optimize these settings. Initial parameters are typically set to an output current of 0.25 mA (and eventually increased to 2–3 mA as tolerated), signal frequency of 30 Hz, pulse width of 250–500 μs, stimulation “on” time 30 s, and stimulation off-time 300 s (16). Traditionally, VNS treatment utilizes the left vagus nerve due to fear for theoretically increasing risk of cardiac side effects. Some evidence suggests that long-term right-sided VNS is actually associated with reactive airway disease and can be considered if left-sided VNS cannot be performed (17). Currently known side effects of VNS, in addition to the involvement of surgery, include cough, hoarseness, voice alteration, and paresthesias (18).

### Ischemic stroke: Relevance and pathophysiology

**Figure 2:**
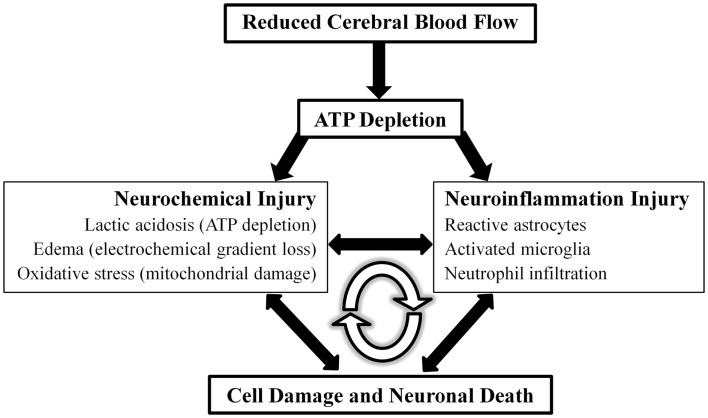
**Model of ischemic stroke acute pathophysiology**. Detrimental acute effects of ischemic stroke can be conceptualized into two separate but highly inter-related physiological entities, neurochemical and neuroinflammation injury, that ultimately lead to cellular damage and death. These detrimental effects function in a positive feedback loop.

Stroke (cerebrovascular disease) is the fourth leading cause of death in the United States, with approximately 795,000 people experiencing a new or recurrent stroke every year (19). Ischemic stroke accounts for more than 80% of stroke that occurs in the United States. Acute neuronal damage from ischemic stroke can be considered to be generated from two main mechanisms: neurochemical changes and neuroinflammatory injury (Figure 2).

The current model for acute neurochemical changes after ischemic stroke describes decreased perfusion and ATP depletion as the cause of electrochemical gradient disruption, release of neurotransmitters (e.g., glutamate excitotoxicity), cytotoxic edema, oxidative stress, and cell death pathways (20). Neuroinflammatory injury is dependent on inflammatory cytokines and adhesion molecules that recruit neutrophils, macrophages, and activate microglia. The combination of neurochemical and neuroinflammatory injury leads to endothelial damage and failure of the blood–brain barrier, which results in intracerebral hemorrhage and edema (21). These deleterious events can be conceptualized as a positive feedback loop. This study looks to further examine evidence for the role of VNS in the setting of ischemic stroke in the current literature and possible mechanisms of action to explain the observed results.

## Methods

**Figure 3:**
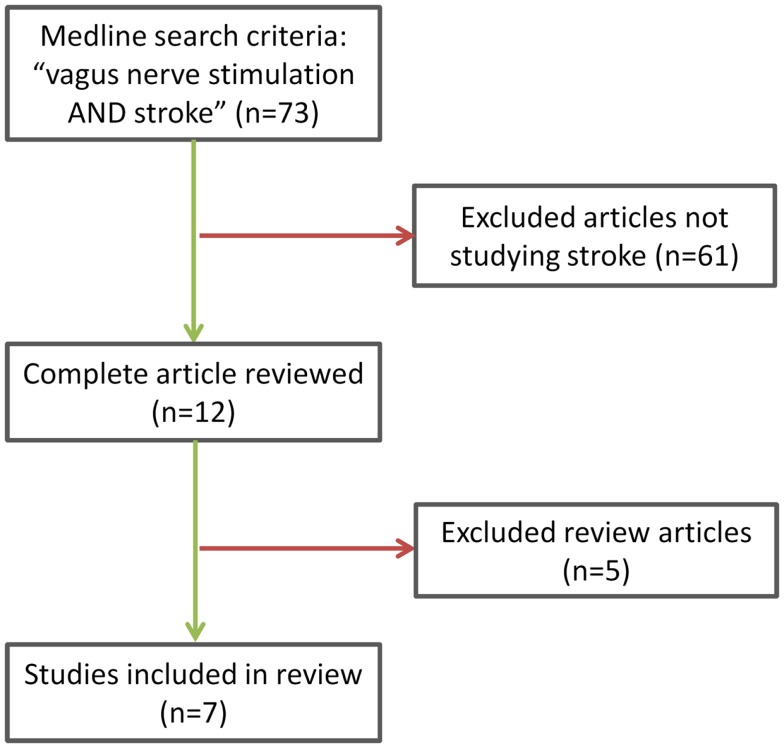
**Study inclusion criteria methodology**.

A systematic search was performed for publications in Medline (1966–2014) with keywords “VNS AND stroke” in subject headings and key words with no language restrictions. Of the 73 publications retrieved, we identified 7 studies from 3 different research groups that met our final inclusion criteria (Figure 3) of research studies addressing the role of VNS in ischemic stroke. Two reviewers, PC and AB, independently selected the relevant studies and discrepancies about inclusion were resolved by VH. Studies included were all experimental animal studies because VNS remains a novel treatment idea with no clinical data to date. An additional search of references using Highwire resulted in two additional relevant abstracts but no available data were provided.

## Results

Our search yielded seven studies that have examined the effect of VNS on improving outcomes, as measured by neurological deficit score (NDS), stroke volume, forelimb strength, and a bradykinesia assessment task after various rat ischemic stroke models (22–28). Detailed study characteristics are listed in Table 1. Five studies stimulated the right vagus nerve 30 min after ischemia at 5-min intervals for a total of 60 min in rats. In these studies, the same stimulation strength and frequencies were used but the duration varied between either 0.5 or 0.3 ms. Two additional studies stimulated the right vagus nerve over a period of 25 days during rehabilitation training. One study included a left VNS protocol with 105 min occlusion of the right middle cerebral artery (23) and a third group included a pdMCAO model using photochromatic occlusion (25).

**Table 1:** **Detailed study characteristics and treatment**.

Study	Study groups	Ischemia model	VNS stimulation	VNS duration
Ay et al. (22)	Right VNS experiment gr 1 Right VNS experiment gr 2 Control gr (*n* = 6 in each gr)	Right TMCAO (120 min)	^a^30 min after ischemia; 0.5 ms EG1: stimulation every 30 min EG2: stimulation every 5 min	3 h
	Summary: right VNS reduced infarct size and improved functional scores with two different stimulation protocols after TMCAO			
Ay et al. (23)	Right VNS experiment gr Control gr (*n* = 8 in each gr)	Right TMCAO (120 min – right VNS)	^a^30 min after ischemia; 0.5 ms stimulation every 5 min	1 h
	Left VNS experiment gr	Right TMCAO (105 min – left VNS) Control gr (*n* = 8 in each gr)	^a^30 min after ischemia; 0.5 ms stimulation every 5 min	1 h
	Summary: both right and left VNS reduced infarct size and improved functional scores			
Ay and Ay (26)	Right VNS experiment gr with intact SPG Right VNS experiment gr with SPG ablation	Right TMCAO (120 min – right VNS)	^a^30 min after ischemia; 0.5 ms stimulation every 5 min	1 h
	Summary: right VNS reduced infarct size and improved functional scores in SPG-intact and SPG-damaged animals			
Hiraki et al. (24)	Right VNS experiment gr (*n* = 10) Control gr (*n* = 10) Sham (*n* = 8)	Right TMCAO (120 min)	^a^30 min after ischemia; 0.3 ms stimulation every 5 min	1 h
	Summary: right VNS reduced infarct size and improved functional scores			
Sun et al. (25)	Right VNS experiment gr Control group (*n* = 8 in each group)	Right TMCAO (120 min)	^a^30 min after ischemia; 0.3 ms stimulation every 5 min	1 h
	Right VNS experiment gr Control gr (*n* = 8 in each group)	Right PMCAO with photochromatic occlusion	^a^30 min after ischemia; 0.3 ms stimulation every 5 min	1 h
	Summary: right VNS reduced infarct size after both TMCAO and PMCAO but only improved functional scores after TMCAO and not PMCAO			
Khodaparast et al. (27)	Left VNS experiment gr (*n* = 6) Control group (*n* = 9)	Endothelin-1 injection at forelimb area of primary motor cortex	^b^50 ms within successful pull attempt; 0.1 ms stimulation, 15 pulses over 500 ms	25 days
	Summary: left VNS paired with rehabilitative training restored forelimb strength to pre-lesion performance			
Khodaparast et al. (28)	Left VNS experiment gr (*n* = 8) Control group (*n* = 9)	Endothelin-1 injection at forelimb area of primary motor cortex	^b^50 ms within successful pull attempt; 0.1 ms stimulation, 15 pulses over 500 ms	25 days
	Summary: left VNS paired with rehabilitative training restored forelimb function to pre-lesion performance			

*MCAO, middle cerebral artery occlusion; VNS, vagus nerve stimulation; gr, group; TMCAO, temporary middle cerebral artery occlusion; PMCAO, permanent middle cerebral artery occlusion; SPG, sphenopalatine ganglion*.

*^a^ Groups used 0.5 mA and 20 Hz pulse trains*.

*^b^ Groups used 0.8 mA and 30 Hz pulse trains*.

Of the studies that measured infarct volume, four performed staining of 2 mm sections of unfixed brain tissue with 2% 2,3,5-triphenyltetrazolium chloride (TTC) (22, 23, 25, 26). One study used overnight fixation of 10-μm sections of brain tissue stained with hematoxylin–eosin (HE) (24). Previous data in animal models of MCAO suggested that TTC and HE staining are significantly correlated when quantifying ischemic injury (29, 30). Overall, a significant decrease in infarct volume was seen in all experimental groups given VNS as compared to control groups following induction of ischemic stroke. All studies showed significant decrease in infarct volume in VNS treatment groups. One group also examined the role of the sphenopalatine ganglion (SPG), and the possible involvement of the parasympathetic vasodilator fibers to the anterior cerebral circulation, on VNS-mediated improvements in outcome following ischemic stroke. Both SPG-intact and SPG-damaged animals treated with VNS demonstrated reduced infarct volume and improved motor outcome when compared to controls (26). Several of the studies measured neurological deficit on a 5-point scale following observation for forelimb flexion, resistance to lateral movement, and circling behavior 24 h after ischemia (22, 23). One study used a 12-point scale based on sensorimotor measurements (25). All experiments showed a statistically significant improvement in NDS of the VNS treatment groups after ischemic stroke in comparison to the control groups. Finally, one group found that subsequent to rat ischemic stroke in the primary motor cortex, VNS during rehabilitative training restored forelimb strength and bradykinesia assessment task to pre-lesion levels while rehabilitative training alone failed to restore function to pre-lesion levels (27, 28).

## Discussion

In this review of experimental studies, VNS showed consistent favorable effects on outcome in various rat ischemic stroke models. One commonly used model was TMCAO (transient middle cerebral artery occlusion), which is especially relevant for translation into clinical application because occlusion of the middle cerebral artery is the leading worldwide cause of ischemic stroke. In comparison to controls, VNS-treated groups demonstrated attenuated infarct size, reduced neurological deficit, and improved forelimb functioning after ischemic stroke.

Current experimental evidence for the role of the vagus nerve in regulating a number of distinct pathways involved in ischemic stroke pathophysiology include: (A) CBF, (B) melanocortins and inflammation, (C) glutamate excitotoxicity, (D) NE, and (E) neurotrophic processes (15).

### Cerebral blood flow

Vagus nerve stimulation used in rats has been shown to decrease CBF during 30 s stimulation periods in an ischemic stroke model and attenuate cerebral edema after brain injury (23, 31). In patients with treatment-refractory major depressive disorder, CBF decreases (left and right lateral orbitofrontal cortex, left inferior temporal lobe) and increases (right dorsal anterior cingulated, left posterior limb of internal capsule/medial putamen, right superior temporal gyrus, left cerebellar body) were seen in different anatomical regions after VNS (32).

### Melanocortins and inflammation

The cholinergic anti-inflammatory pathway is regulated by melanocortin-synthesizing neurons. Increased activity of this pathway was protective while decreased activity has been associated with worse neurological symptoms in ischemic stroke patients (33). In rats, melanocyte-stimulating hormone (NDP-α-MSH) was shown to be neuroprotective after ischemic stroke by suppressing inflammation and apoptotic cascades both centrally and peripherally. Interestingly, bilateral vagotomy appears to blunt the protective effects of NDP-α-MSH administration (34). Animal models suggest that TNF levels are modulated by the cholinergic anti-inflammatory pathway of the vagus nerve (35).

### Glutamate excitotoxicity

In gerbil hippocampus, VNS has been shown to decrease glutamate release in a transient global ischemia stroke model (36). Walker and colleagues characterized the ability for glutamate antagonists to decrease glutamate release in the NTS in helping to block seizures (37). VNS on patients with medically intractable epilepsy also resulted in decreased glutamate levels (38).

### Norepinephrine

Increased extracellular concentration of NE was observed in both the hippocampus and cortex after VNS at the cervical level in rats. Increases in NE were observed during stimulus periods and elevations returned to baseline in inter-stimulus periods. In addition, increased intensity of VNS was associated with increasing levels of NE concentration (39). The effects of NE release were initially studied for its role in suppressing seizures, improving depression, enhancing learning and memory, and improving function after traumatic brain injury. However, the convergence of different pathophysiological mechanisms among these conditions and ischemic stroke suggests a possibility for stroke treatment.

### Neurotrophic processes

Electroconvulsive therapy (ECT) and antidepressant medication have been previously proposed to increase hippocampal brain-derived neurotrophic factor (BDNF) and Fos protein, which promotes survival and growth of neurons in patients (40). Subsequent research a decade later implicated VNS in upregulating gene expression of BDNF and fibroblast growth factor (bFGF) in the rat brain (41), suggesting a possible mechanism for the beneficial effects seen in the treatment of depression with VNS.

We hypothesize that VNS plays a role in ischemic stroke pathogenesis mainly through the mechanisms of attenuating excitotoxicity and inhibiting inflammation in the acute phase and modulating neuroplasticity in the chronic phase. In the pathogenesis of cerebral ischemia, glutamate excitotoxicity occurs in the acute phase (minutes–hours) and inflammation begins in the subacute phase (hours–days) (21). Excessive synaptic release of glutamate can cause glutamate excitotoxicity, which plays a role in stroke, nervous system trauma, epilepsy, and chronic neurodegenerative disorders (42). As mentioned previously, VNS was associated with decreased glutamate release and neuroprotection in the gerbil hippocampus (36). VNS modulates neuroinflammation by two main mechanisms: NE release and activation of the cholinergic anti-inflammatory pathway (33). NE has been shown to be neuroprotective and associated with anti-inflammation, which may be due to its ability to suppress nitric oxide synthase, chemokines, and cell adhesion molecules (43, 44). Reboxetine, a drug known to inhibit reuptake of NE, has demonstrated an ability to improve motor ability in chronic stroke patients (45), suggesting a relationship between the presence of NE and neuroprotection. VNS also activates a cholinergic anti-inflammatory response and may reduce both brain and systemic inflammation. Current understanding suggests the cholinergic anti-inflammatory pathway is driven by the efferent vagus nerve at nAChRα7 receptors (15). Studies demonstrate that stimulation of nAChRα7 receptors attenuates inflammation by regulating microglial activation in the brain and protects neuronal cells from oxidative stress (46, 47). Melanocortins, which function as cholinergic anti-inflammatory pathway regulators, downregulate tumor necrosis factor-α levels after ischemic stroke in a vagus nerve-dependent manner (34). These studies suggest that the vagal cholinergic pathway plays an important role in mediating inflammation after ischemic stroke. Through all of the neuromodulatory effects of VNS, such as release of acetylcholine, NE, and BDNF, it has been previously proposed that VNS may induce plasticity in the motor cortex in the chronic stage (27). However, the cellular and molecular mechanisms behind VNS-dependent neuroplasticity remain unclear.

CNI-1493, or Semapimod, underwent Phase II clinical trials for treatment of Crohn’s disease. This compound is known to inhibit systemic inflammation and was shown to be protective for stroke in preclinical testing. Subsequently, CNI-1493 was also shown to stimulate vagal nerve activity (48). These findings further support the idea that VNS may be protective in stroke through the suppression of inflammation.

Interestingly, a non-invasive transcutaneous VNS (T-VNS), which stimulates the auricular branch of the vagus nerve, appeared on the European market in 2012 for seizure frequency reduction. T-VNS has been shown to be safe and tolerable in a retrospective and pilot study (49, 50). Initial treatment with T-VNS in one proof of concept trial included stimulation for 1 h in the morning, 1 h at noon, and 1 h in the evening with the following settings: stimulation frequency of 10 Hz, pulse width of 300 μs, applied voltage of about 25 V (adjusted based on individual patient tolerance), and stimulation area of 2 cm^2^ (50). These parameters vary in different trials as different settings are being studied. This treatment modality has also been studied in a rat seizure model, which describes a relationship between the auricular branch of the vagus and the autonomic and central nervous system (51). While these studies support the possibility for T-VNS as an effective alternative, it remains to be seen whether this non-invasive approach to VNS is equally as effective. Because traditional VNS implantation involves surgery, its clinical application may pose some patients’ safety problems in the setting of an acute stroke. Hence, non-invasive VNS appears to be a more practical and safe option. Initial studies using T-VNS in pain perception and epilepsy suggest that there may be an association with mild ulceration of the skin at the stimulation area but no severe side effects (52, 53).

There are several limitations with the literature we have reviewed. First, because this is a novel topic, the literature does not provide a universally accepted mechanism for the role of VNS in brain ischemia and more animal work should be done to further elucidate the mechanism. Due to this new topic in the field of ischemic stroke research, our speculations are appropriately wide-ranging. Also, the seven studies included in our review are derived from three research groups, which may impose certain biases to the results. Finally, the utility of and translation of rat stroke pathogenesis for human stroke outcomes is questionable because differences in mouse genetics, anatomy, and physiology may all influence the mechanisms associated with stroke tissue necrosis and outcomes (54–56). For example, animal ischemic stroke models are carried out on healthy animals whereas stroke patients often have a host of other comorbidities (aging, hypertension, diabetes, heart disease, and medications) (57). Also, since stimulation of the vagus nerve begins after the ischemia, it is not due to ischemic preconditioning and future animal experiment methodology should strive to better simulate clinical experience as starting therapy 30 min from stroke onset may not be clinically translatable. Finally, our data analysis is limited by the heterogeneity of studies, such as surgical techniques, stroke volume data reporting, and behavioral testing.

## Conclusion

In experimental stroke models, VNS attenuates ischemic stroke volume, reduces neurological deficits, and improves forelimb functioning. Currently, none of 19 neuroprotection Phase III trials analyzed were shown to have positive outcomes (58). VNS may be a promising therapy that targets many different neuroprotective pathways and should be studied for the treatment of post-ischemic stroke. Past clinical experience with VNS treatment confirms its safety and efficacy with only mild to moderate side effects that are predictable and shown to improve over time (18). In addition, the convenience and low morbidity of using the newly developed T-VNS modality is encouraging for future clinical studies. Given its efficacy in stroke models, its establishment as a safe treatment modality for other conditions, and the convenience of new technological developments, we believe it is valid to further examine the role of VNS as a neuromodulator in both acute and chronic phase of clinical stroke as well as a possible secondary prophylactic option.

## Author Contributions

Study concept and design: Cai, Bodhit, Hedna. Acquisition of data: Cai, Bodhit, Derequito, Ansari, Abukhalil, Thenkabail, Ganji, Saravanapavan, Shekhar, Bidari, Waters, Hedna. Analysis and interpretation of data: Cai, Bodhit, Derequito, Ansari, Hedna. Drafting and critical revision of manuscript: Cai, Bodhit, Derequito, Ansari, Abukhalil, Thenkabail, Ganji, Saravanapavan, Shekhar, Bidari, Waters, Hedna. Statistical analysis: Cai, Bodhit. Administrative, technical, and material support: Waters, Hedna. Study supervision: Waters, Hedna.

## Conflict of Interest Statement

The authors declare that the research was conducted in the absence of any commercial or financial relationships that could be construed as a potential conflict of interest.
